# T cells in autoimmune thyroid disease and pregnancy: an emerging immunological interface

**DOI:** 10.3389/fimmu.2026.1958382

**Published:** 2026-10-22

**Authors:** Ivona Perić, Leo Fischer, Maja Ilić Tomaš, Igor Iskra, Ines Krištofić, Vlatka Sotošek, Lara Batičić, Sanja Klobučar, Tatjana Bogović Crnčić

**Affiliations:** 1 Department of Endocrinology, Diabetes and Metabolic Diseases, Clinical Hospital Centre Rijeka, Rijeka, Croatia; 2 Department of Nuclear Medicine, Faculty of Medicine, University of Rijeka, Rijeka, Croatia; 3 Clinical Department of Nuclear Medicine, Clinical Hospital Centre Rijeka, Rijeka, Croatia; 4 Department of Obstetrics and Gynecology, Faculty of Medicine, University of Rijeka, Rijeka, Croatia; 5 Department of Anesthesiology, Reanimatology and Intensive Care Medicine, Faculty of Medicine, University of Rijeka, Rijeka, Croatia; 6 Department of Clinical Medical Sciences II, Faculty of Health Studies, University of Rijeka, Rijeka, Croatia; 7 Clinic of Anesthesiology, Intensive Care Medicine and Pain Management, Clinical Hospital Centre Rijeka, Rijeka, Croatia; 8 Department of Medical Chemistry, Biochemistry and Clinical Chemistry, Faculty of Medicine, University of Rijeka, Rijeka, Croatia; 9 Department of Internal Medicine, Faculty of Medicine, University of Rijeka, Rijeka, Croatia

**Keywords:** autoimmunity, immune tolerance, pregnancy, Th17 cells, thyroiditis, T-lymphocytes

## Abstract

Pregnancy is a condition that requires highly coordinated immunological adaptations to ensure maternal–fetal tolerance while preserving effective immune defense. Among adaptive immune cells, T lymphocytes play a central role in regulating the balance between immune activation and immune tolerance throughout gestation. Distinct T-cell subsets—including T helper (Th)1, Th2, Th17, regulatory T (Treg), and T follicular helper (Tfh) cells—contribute to the dynamic immune changes necessary for a successful pregnancy. Autoimmune thyroid disease (AITD), encompassing Hashimoto’s thyroiditis and Graves’ disease, is characterized by dysregulated T-cell–mediated immune responses, including enhanced Th1 and Th17 activity and impaired Treg-mediated immune regulation. As AITD frequently affects women of reproductive age, the interaction between AITD and pregnancy-related immune adaptation represents a clinical and immunological challenge. Increasing evidence suggests that persistent immune dysregulation may contribute to thyroid dysfunction and adverse pregnancy outcomes, including pregnancy loss, preterm birth, preeclampsia, and fetal growth restriction. This review summarizes current evidence on T-cell–mediated immune regulation in normal pregnancy and AITD, with particular emphasis on the immunological interactions between these conditions. We discuss physiological changes in major T-cell subsets during pregnancy, mechanisms of immune dysregulation in AITD, and their potential implications for maternal and fetal outcomes. The potential roles of T-cell memory and postpartum immune changes are also briefly considered. A better understanding of T-cell dynamics at the intersection of pregnancy and thyroid autoimmunity may provide further insight into underlying immunological mechanisms and, with further validation, potentially inform future approaches to risk stratification and individualized management.

## 1 Introduction

Pregnancy is a unique immunological condition in which the maternal immune system must tolerate the semi-allogeneic fetus while maintaining effective defense against pathogens. This paradox represents a central question in reproductive immunology and reflects the complex, dynamic interactions between maternal immune cells and fetal tissues. Rather than a state of generalized immune suppression, pregnancy is now recognized as a tightly regulated immunological process characterized by spatial and temporal modulation of immune responses throughout gestation (1).

Successful pregnancy relies on finely tuned communication at the maternal–fetal interface, where both innate and adaptive immune cells contribute to fetal tolerance and tissue remodeling. Among adaptive immune cells, T lymphocytes are essential for maintaining the balance between immune tolerance and protective immunity (2, 3). Distinct CD4^+^ T-cell subsets, including T helper (Th)1, Th2, Th17, and regulatory T (Treg) cells, demonstrate functional plasticity and are considered crucial for preserving this equilibrium during pregnancy (2–4).

Autoimmune thyroid disease (AITD), primarily encompassing Hashimoto’s thyroiditis (HT) and Graves’ disease (GD), is among the most common organ-specific autoimmune disorders and is characterized by dysregulated cellular and humoral immune responses targeting thyroid antigens (4, 5). The pathogenesis of AITD is centered on the activation and infiltration of autoreactive T lymphocytes, along with an imbalance in T-cell subsets, such as increased pro-inflammatory Th1 and Th17 responses and impaired regulatory T-cell function (4, 6). Reduced Treg numbers and phenotypic abnormalities further contribute to the breakdown of immune tolerance in AITD (6, 7).

AITD is common among women of reproductive age and has been associated with adverse pregnancy outcomes, including pregnancy loss, preterm birth, preeclampsia, and fetal growth restriction (8, 9). Although thyroid hormone imbalance contributes to these complications, immune dysregulation has been proposed as an additional potential mechanism linking thyroid autoimmunity with adverse pregnancy outcomes (8, 9). Furthermore, pregnancy induces profound immunological changes that may transiently modulate autoimmune responses but can also lead to disease exacerbation in the postpartum period (2, 10, 11).

As both pregnancy and AITD involve significant modulation of T-cell–mediated immunity, their interaction represents a growing and clinically significant immunological interface. Pregnancy and AITD may reflect partially opposing immunological states: tolerance versus dysregulated immune activation, placing T-cell dynamics at the center of this interaction. Therefore, dysregulation within specific T-cell subsets may critically influence whether physiological adaptation or pathological processes predominate during gestation.

Although T-cell dysregulation has been described in AITD and T-cell imbalance has been associated with adverse pregnancy outcomes in broader reproductive populations, the extent to which these processes intersect in pregnancies complicated by AITD remains incompletely understood. Existing reviews have largely addressed T-cell immunity in pregnancy, AITD immunopathogenesis, and thyroid autoimmunity in pregnancy as related but distinct topics. However, the specific intersection between pregnancy-associated T-cell adaptation and T-cell dysregulation in AITD, including the extent to which current evidence directly supports their involvement in pregnancy outcomes, has received less focused attention.

This narrative mini-review therefore aims to integrate current evidence across these areas, with particular emphasis on the pregnancy–AITD T-cell interface, while distinguishing established observations from emerging and indirect evidence.

The major T-cell subsets discussed throughout this review, together with their physiological roles during pregnancy, immune alterations in AITD, and potential implications for pregnancy outcomes, are summarized in **Table 1**.

**Table 1:** Functional characteristics of major T-cell subsets in normal pregnancy, reported alterations in autoimmune thyroid disease, and evidence context for potential pregnancy implications.

T-cell subset	Physiological role in pregnancy	Reported immune alterations and AITD phenotype	Potential pregnancy implications and evidence context
Th1	Supports implantation and placentation during early pregnancy; contributes to tissue remodeling (3, 32)	Prominent Th1-mediated responses in HT; increased IFN-γ and IL-2 during pregnancy in both GD and HT (13, 27)	Direct human evidence in pregnant women with GD and HT indicates that higher IFN-γ and IL-2 levels are associated with adverse pregnancy outcomes (13). Indirect evidence from the broader reproductive literature suggests that dysregulated Th1/Th2 responses are also associated with recurrent pregnancy loss and other adverse pregnancy outcomes (3).
Th2	Promotes anti-inflammatory immune environment and maternal–fetal tolerance (2)	Reduced IL-4 and IL-10 during pregnancy in both GD and HT, consistent with relative Th1 predominance (13)	Direct human evidence in pregnant women with GD and HT indicates that lower IL-4 and IL-10 levels are associated with adverse pregnancy outcomes (13).
Treg	Maintains maternal–fetal tolerance and suppresses autoreactive immune responses (18, 25)	Reduced Treg frequency/number has been reported in both GD and HT (6), including during pregnancy (13); additional Treg-subset abnormalities have been described specifically in HT (7).	Indirect evidence from the broader reproductive and obstetric literature indicates that reduced Treg number and/or function is associated with recurrent pregnancy loss, implantation failure, and preeclampsia (15, 42–45).
Th17	Participates in host defense and inflammatory responses (17)	IL-17/Th17 pathway involvement has been reported in both GD and HT, although the patterns may differ according to disease phenotype (29).	Human evidence indicates that TPOAb-positive women with recurrent pregnancy loss exhibit increased Th17 activity and reduced Treg-associated markers (46). Indirect evidence from broader obstetric populations further implicates Th17/Treg imbalance in preeclampsia (44).
Tfh	Regulates B-cell activation and antibody production (23)	Role in AITD remains insufficiently characterized, particularly during pregnancy.	Experimental evidence indicates that dysregulated Tfh responses are associated with fetal resorption in murine pregnancy models, whereas direct evidence in pregnancies complicated by AITD is currently lacking (24). *
Memory T cells	Contribute to long-term immune adaptation following pregnancy, with a possible role in immune tolerance in subsequent pregnancies (11, 54).	The specific role in GD and HT remains largely unexplored.	Human evidence from non-AITD populations suggests that pregnancy-related immune-memory changes may persist beyond pregnancy, but AITD-specific pregnancy evidence is lacking (54, 56) *

*Evidence remains limited and requires further investigation.

The table summarizes the principal T-cell subsets involved in physiological pregnancy, reported immune alterations in AITD, and their potential pregnancy implications. Where supported by the source data, immune alterations are specified according to AITD phenotype (Hashimoto’s thyroiditis or Graves’ disease). The evidence context for pregnancy implications is indicated within each row according to the population and type of evidence, distinguishing direct human evidence in pregnant women with defined AITD, human evidence from thyroid-autoimmunity populations, indirect evidence derived from broader reproductive or obstetric literature, and experimental evidence. These categories describe the source and context of the reported associations and should not be interpreted as establishing causality. Where AITD-specific pregnancy evidence is unavailable or insufficient, this is explicitly stated. Direct evidence concerning Tfh and memory T cells in pregnancies complicated by AITD remains particularly limited.

AITD, autoimmune thyroid disease; HT, Hashimoto’s thyroiditis; GD, Graves’ disease; TPOAb, thyroid peroxidase antibodies; IFN-γ, interferon gamma; IL-2, interleukin 2; IL-17, interleukin 17; Tfh, T follicular helper cells; Th1, T helper 1 cells; Th2, T helper 2 cells; Th17, T helper 17 cells; TNF-α, tumor necrosis factor alpha; Treg, regulatory T cells.

## 2 Methods

Literature for this narrative Mini Review was identified primarily through searches of PubMed initiated in April 2026, supplemented by screening the reference lists of relevant articles. Search terms included combinations of “autoimmune thyroid disease”, “Hashimoto’s thyroiditis”, “Graves’ disease”, “pregnancy”, “T cells”, “Th1”, “Th2”, “Th17”, “regulatory T cells”, “T follicular helper cells”, and “memory T cells”. Priority was given to studies directly relevant to T-cell immunity in pregnancy and AITD, while selected studies from reproductive immunology and experimental models were included where direct evidence in pregnant women with AITD was limited. The literature search was last updated in July–August 2026. Given the narrative nature of this Mini Review, the search was intended to provide a focused synthesis rather than a systematic or exhaustive review of the literature.

## 3 Immune adaptations during normal pregnancy

Pregnancy is not a state of generalized maternal immunosuppression, but rather a highly coordinated immunological process characterized by dynamic and temporally regulated adaptations of both innate and adaptive immunity. A widely accepted concept is the triphasic model of pregnancy immunity, which describes an initial pro-inflammatory phase associated with implantation and placentation, followed by a predominantly regulatory and anti-inflammatory phase during fetal growth, and finally a renewed inflammatory phase preceding labor and parturition. These immunological shifts allow maintenance of maternal–fetal tolerance while preserving adequate host defense against pathogens (12).

Successful pregnancy depends on complex interactions between trophoblasts and maternal immune cells at the maternal–fetal interface, particularly decidual natural killer (dNK) cells, macrophages, antigen-presenting cells, and Treg cells (1, 10). Regulatory T cells represent a specialized subset of CD4^+^ T lymphocytes responsible for maintaining immune tolerance through cell-to-cell contact and secretion of inhibitory cytokines, including interleukin-10 (IL-10) and transforming growth factor beta (TGF-β) (13, 14). Although multiple Treg subpopulations have been identified, CD4+CD25+ T cells expressing forkhead box P3 (FOXP3) remain the best-characterized Treg subset in pregnancy immunology (7). Extravillous trophoblast cells express polymorphic human leukocyte antigen C (HLA-C) molecules, which participate in interactions with maternal decidual immune cells, particularly through maternal killer-cell immunoglobulin-like receptor (KIR)–fetal HLA-C combinations. Maternal T-cell tolerance also involves direct and indirect presentation of paternal and fetal antigens (10, 14). CD4^+^CD25^+^FOXP3^+^ Tregs are considered key mediators of maternal–fetal tolerance in both humans and experimental models (10, 13–15).

Rather than representing a simple switch from Th1 to Th2 immunity, pregnancy involves compartment-specific and time-dependent modulation of immune responses. Adaptive immunity gradually shifts toward a Th2- and Treg-favoring environment, whereas excessive Th1-mediated cytotoxic inflammation is restrained to preserve fetal tolerance. At the same time, dNK cells acquire predominantly immunoregulatory and vascular-remodeling functions instead of classical cytotoxic activity (12, 16, 17).

The first trimester is characterized by a carefully regulated inflammatory environment required for implantation, trophoblast invasion, and spiral artery remodeling. Cytokines such as tumor necrosis factor alpha (TNF-α), interleukin-1 beta (IL-1β), interferon gamma (IFN-γ), and interleukin-8 (IL-8) contribute to tissue remodeling and angiogenesis, while anti-inflammatory mediators including IL-10, TGF-β, and indoleamine 2,3-dioxygenase (IDO) simultaneously limit excessive immune activation and promote tolerance (12, 14, 16). Decidual leukocytes are particularly abundant during early pregnancy, with dNK cells representing the predominant immune cell population (12, 16). In parallel, Tregs expand early in gestation and play a central role in suppressing maternal anti-fetal immune responses through direct cellular interactions and secretion of inhibitory cytokines such as IL-10 and TGF-β (12, 17–19).

During the second trimester, immune regulation becomes more pronounced and maternal–fetal tolerance reaches its functional peak (12, 16, 17). This phase is characterized by relative predominance of Th2- and Treg-associated pathways together with reduced inflammatory activity. Cytokines such as IL-4, IL-5, IL-10, and IL-13 contribute to maintenance of an anti-inflammatory environment, while sustained FOXP3 expression and tolerogenic antigen presentation support Treg stability and suppress fetal antigen-reactive effector T cells (16–20). Rather than completely suppressing Th1 immunity, pregnancy appears to involve a balanced control of inflammatory responses.

In late pregnancy, inflammatory pathways are gradually reactivated in preparation for labor. Increased production of IL-1β, IL-6, IL-8, and TNF-α contributes to cervical ripening, membrane activation, leukocyte recruitment, and myometrial contractility (12, 21). Nevertheless, maternal–fetal tolerance must remain preserved until delivery, and Tregs continue to play an important role in controlling excessive anti-fetal immune responses during this period. Therefore, the third trimester represents a state of controlled inflammatory reactivation rather than complete reversal toward a classical Th1-dominant immune profile (22).

Furthermore, Th17 lymphocytes represent a subset of CD4^+^ T-helper cells that characteristically produce IL-17A and IL-17F and may also secrete IL-21, IL-22, and other inflammatory mediators, depending on the local cytokine environment (17). Th17 differentiation and stabilization are regulated by a cytokine network involving TGF-β, IL-6, IL-1β, IL-21, and IL-23, with retinoic acid receptor-related orphan receptor gamma t (ROR-γt) acting as a key lineage-associated transcription factor. Although Th17 cells participate in physiological immune adaptation during pregnancy, excessive Th17 activation has been associated with recurrent pregnancy loss and other adverse pregnancy outcomes (2). Tfh cells regulate B-cell activation and antibody production and are increasingly recognized as important modulators of immune adaptation during pregnancy (23, 24). Human studies have demonstrated pregnancy-associated expansion of circulating functional Tfh cells (23), whereas experimental murine studies have associated enhanced Tfh accumulation with increased fetal resorption following programmed death-ligand 1 (PD-L1) blockade (24).

Pregnancy-associated hormonal changes further contribute to immune adaptation. Elevated estradiol, progesterone, and human chorionic gonadotropin levels promote Th2 polarization, enhance Treg expansion, and suppress excessive Th1-mediated immune activation (12, 25). These endocrine effects interact closely with tolerogenic antigen presentation and regulatory immune pathways, collectively supporting maternal–fetal tolerance throughout gestation (16, 25).

Taken together, normal pregnancy represents a highly specialized immunological state characterized by tightly coordinated interactions between inflammatory and regulatory pathways. Rather than generalized immune suppression, successful gestation depends on dynamic, trimester-specific modulation of T-cell responses that balances fetal tolerance with preservation of maternal immune competence (12, 16, 25). Disruption of these tightly regulated immune mechanisms may contribute not only to pregnancy complications but also to the development or exacerbation of autoimmune disorders, including AITD (2).

## 4 T-cell dysregulation in autoimmune thyroid disease

AITD encompasses several autoimmune conditions, primarily Hashimoto’s thyroiditis and Graves’ disease, which are among the most common organ-specific autoimmune disorders characterized by loss of immune tolerance toward thyroid antigens. Although genetic susceptibility contributes to disease development, current evidence suggests that dysregulation of cellular immunity, particularly T-cell-mediated immune responses, represents a central mechanism in the pathogenesis of thyroid autoimmunity (26, 27). Although Hashimoto’s thyroiditis and Graves’ disease share several T-cell abnormalities, they represent distinct immunological and clinical phenotypes. Hashimoto’s thyroiditis is more strongly associated with destructive, predominantly Th1-mediated cellular responses, whereas Graves’ disease is characterized by thyroid-stimulating hormone receptor (TSHR)-stimulating autoantibodies and prominent T-cell–B-cell interactions. Therefore, where supported by the available evidence, findings are specified according to disease phenotype.

The thyroid gland in AITD is infiltrated by autoreactive T lymphocytes, including CD4^+^ helper T cells and CD8^+^ cytotoxic T cells, which promote chronic inflammation and progressive thyroid dysfunction (27). Antigen-presenting cells activate naïve CD4^+^ T lymphocytes after exposure to thyroid autoantigens such as thyroid peroxidase (TPO), thyroglobulin (Tg), and TSHR. Activated T cells subsequently differentiate into distinct functional subsets, including Th1, Th17, and Treg cells, whose imbalance may contribute significantly to disease progression (26, 27).

Th1-mediated immune responses are strongly associated with thyroid tissue injury, particularly in Hashimoto’s thyroiditis. Th1 lymphocytes produce pro-inflammatory cytokines such as IFN-γ, IL-2, and TNF-α, which stimulate macrophage activation, enhance antigen presentation, and promote apoptosis of thyroid follicular cells. Elevated circulating levels of IFN-γ and IL-2 have been reported in AITD, including in patients with Graves’ disease, although findings vary according to disease phenotype, activity, and analytical method (27, 28).

Recent studies have highlighted the importance of Th17 lymphocytes and IL-17 signaling pathways in AITD. Th17 cells produce IL-17A, IL-17F, IL-21, and IL-22, which amplify inflammatory reactions and contribute to recruitment of immune cells into thyroid tissue. Increased Th17 activity and elevated IL-17 expression have been associated with disease severity, enhanced autoantibody production, and progression of thyroid dysfunction. He et al. (29) proposed that the Th17/IL-17 axis may represent a potential therapeutic target in autoimmune thyroid diseases because of its role in sustaining chronic inflammation. However, this mechanistic observation should not be interpreted as support for IL-17-directed treatment during pregnancy, for which efficacy and safety in AITD have not been established.

In contrast, regulatory T cells are essential for maintaining peripheral immune tolerance and suppressing autoreactive immune responses. Reduced Treg frequency and disturbances in the Th17/Treg balance have been reported in both Graves’ disease and Hashimoto’s thyroiditis, supporting a role for defective immune regulation in AITD (6, 26).

Hu et al. (7) additionally reported altered expression of Helios and programmed cell death protein 1 (PD-1) in Treg subsets of patients with Hashimoto’s thyroiditis, suggesting defective immunoregulatory signaling and impaired immune homeostasis. Given the inhibitory role of the PD-1/PD-L1 pathway in limiting T-cell activation and supporting Treg-mediated immune regulation, altered PD-1 expression may contribute to impaired Treg-mediated immune regulation and disrupted immune tolerance; however, the relevance of these alterations during pregnancy remains to be established (7). Alterations in CD4^+^/CD8^+^ T-cell ratios have also been described in AITD (13, 30). Dysregulated helper and cytotoxic T-cell responses may contribute to persistent inflammatory activity and direct thyrocyte injury. Furthermore, these immune abnormalities may interfere with normal pregnancy-related immune adaptations and potentially contribute to adverse pregnancy outcomes in women with thyroid autoimmunity (13, 31).

Environmental and nutritional factors may additionally modulate T-cell responses in AITD. Excess iodine intake has been linked to enhanced activation of pro-inflammatory cytokine pathways and increased Th17-mediated immune responses, while selenium and other micronutrients may influence immune regulation and Treg function (26, 28).

Overall, current evidence indicates that autoimmune thyroid disease is characterized by complex disturbances in T-cell-mediated immunity involving enhanced Th1 and Th17 inflammatory pathways together with impaired Treg-mediated immune tolerance. A deeper understanding of these cellular immune mechanisms may contribute to the development of novel targeted immunomodulatory therapies for AITD.

## 5 Pregnancy-associated immune modulation in women with AITD

### 5.1 Immune adaptation and T-cell dynamics in pregnancies complicated by AITD

Pregnancy is characterized by tightly regulated immunological adaptations, with each stage of gestation requiring a distinct immune environment that supports fetal tolerance while preserving maternal immune defense mechanisms (32).

Physiologically, pregnancy involves time-dependent and compartment-specific regulation of Th2-, Treg-, and inflammatory pathways, contributing to maternal–fetal tolerance while preserving necessary immune activity (2, 14). However, altered T-cell regulation has been demonstrated in pregnant women with both Graves’ disease and Hashimoto’s thyroiditis. In the longitudinal study by Zhong et al. (13), IFN-γ and IL-2 levels were increased, whereas IL-4 and IL-10 levels were reduced in both GD and HT compared with healthy pregnant controls throughout early, middle, and late pregnancy and the postpartum period. TGF-β levels, Treg frequencies, and CD4^+^/CD8^+^ ratios were likewise reduced in both disease groups (13). Furthermore, within both the GD and HT groups, adverse pregnancy outcomes were associated with higher IFN-γ and IL-2 and lower IL-4 and IL-10 levels compared with normal pregnancy outcomes (13). These pregnancy-specific findings are consistent with reduced CD4^+^CD25^+^ regulatory T-cell populations previously reported in both GD and HT outside pregnancy (33). Similarly, Iyidir et al. (34) reported increased IL-2 and IL-17 levels in women with newly diagnosed thyroid autoimmunity during the first trimester compared with healthy pregnant controls, consistent with enhanced Th1- and Th17-associated activity early in pregnancy; these differences were not observed in the second trimester.

Importantly, Th1-related immune activity is not inherently detrimental during pregnancy. Controlled pro-inflammatory responses are essential during implantation and early placentation, where cytokines such as TNF-α and IFN-γ contribute to trophoblast invasion and vascular remodeling (35). However, persistent or excessive Th1 activation beyond early pregnancy may disrupt maternal–fetal immune balance and contribute to pathological pregnancy outcomes (32, 35).

### 5.2 T-cell subsets and adverse pregnancy outcomes

Accumulating evidence indicates that AITD is associated with increased risks of pregnancy loss, preterm birth, preeclampsia, and fetal growth restriction (36–38). Sayed et al. (39) reported an absolute increase in preterm birth risk of approximately 9–18% and nearly fourfold higher odds of preterm delivery in women with thyroid autoimmunity compared with healthy pregnant women.

Immune dysregulation has been proposed as a potential mechanistic link between thyroid autoimmunity and adverse obstetric outcomes; however, direct evidence demonstrating that specific T-cell abnormalities mediate AITD-associated pregnancy complications remains limited. Indirect evidence from broader reproductive and obstetric populations indicates that T-cell abnormalities are associated with adverse pregnancy outcomes. Dysregulated Th1/Th2 responses have been described in recurrent pregnancy loss, whereas alterations in peripheral T-cell populations have been reported in recurrent pregnancy loss and fetal growth restriction (3, 40, 41). Th1-biased immune profiles have been reported both in peripheral blood and at the maternal–fetal interface in women with recurrent pregnancy loss, including increased Th1/Th2 cytokine ratios in peripheral blood (3).

Treg cells represent a central component of maternal–fetal immune tolerance. Expansion and accumulation of Tregs at the maternal–fetal interface suppress autoreactive and effector T-cell responses through direct cellular interactions and secretion of anti-inflammatory cytokines such as IL-10 and TGF-β (18, 25, 42). In the broader reproductive immunology literature, reduced Treg number and impaired suppressive function have been associated with recurrent pregnancy loss, implantation failure, and preeclampsia (42–45). Moreover, circulating Treg frequencies have been investigated as candidate biomarkers of miscarriage risk; however, larger validation studies are required before their clinical utility can be established (45).

Emerging evidence additionally implicates Th17 cells in adverse pregnancy outcomes associated with thyroid autoimmunity. Increased Th17 activity, accompanied by reduced FOXP3 and TGF-β expression, has been reported in women with recurrent pregnancy loss and thyroid autoimmunity (46). Furthermore, Wang et al. (47) demonstrated significant Th17/Treg imbalance in pregnant women with hypothyroidism during the first half of pregnancy, characterized by elevated Th17 cell proportions, reduced Treg cells, and increased levels of IL-2, IL-6, and TNF-α; however, this population should not be considered equivalent to women with defined AITD. Disturbances in the Th17/Treg axis have also been increasingly recognized in the pathogenesis of preeclampsia (44).

Overall, these findings support a biologically plausible association between T-cell dysregulation, thyroid autoimmunity, and adverse pregnancy outcomes. However, direct evidence demonstrating that T-cell abnormalities mediate the increased risk of pregnancy complications specifically associated with AITD remains limited.

### 5.3 Postpartum immune changes

Pregnancy-associated hormonal changes, including elevated estrogen, progesterone, and human chorionic gonadotropin levels, promote Treg expansion and contribute to a more tolerogenic immune profile during gestation (42, 48, 49). Following delivery, withdrawal of pregnancy-associated hormonal and immunological signals is accompanied by immune reconstitution, which has been implicated in postpartum autoimmune activity (42, 48). Postpartum thyroiditis and relapse or exacerbation of pre-existing GD represent clinically distinct manifestations of postpartum thyroid autoimmunity (50, 51). However, direct longitudinal evidence characterizing T-cell subset changes from pregnancy into the postpartum period specifically in women with defined AITD remains limited.

### 5.4 Emerging role of memory T cells and long-term immune adaptation

Pregnancy immunology involves highly coordinated interactions between maternal immune cells, hormonal signals, and fetal antigens that together establish maternal–fetal tolerance. Current evidence on pregnancy-induced memory T-cell responses is derived predominantly from studies of normal pregnancy and experimental models, whereas direct evidence in women with AITD is scarce. Maternal exposure to paternal and fetal antigens before and during pregnancy may induce long-lasting immunological memory responses, including expansion of fetal antigen-specific Treg cells and memory T-cell populations (11, 52, 53). Experimental studies demonstrated that fetal-specific maternal Tregs generated during primary pregnancy may persist after delivery and undergo rapid reactivation in subsequent pregnancies, contributing to more efficient establishment of maternal immune tolerance (52).

Recent studies suggest that pregnancy induces persistent alterations in maternal memory T-cell populations extending beyond the postpartum period. Kieffer et al. (54) demonstrated increased proportions of central and effector memory CD4^+^ T cells in women with previous pregnancies compared with nulligravid women, indicating long-term immunological adaptations induced by gestation. Some epidemiological and experimental observations are compatible with a role for partner-specific immune adaptation in subsequent pregnancies; however, these associations are not sufficient to establish a causal role for T-cell memory (55). Conversely, indirect evidence suggests that pregnancy complications may be associated with persistent alterations in immunological memory, although the clinical significance of these changes for subsequent pregnancies remains uncertain (55, 56).

Recent advances have broadened the conventional Th1/Th2 framework to encompass additional T-cell subsets, such as Tregs, Th17, and Tfh cells, which collectively play important roles in the intricate immune regulation underlying a successful pregnancy. Nevertheless, although increasing evidence indicates that memory T-cell adaptations are involved in pregnancy-related immune changes, their specific contribution to pregnancies affected by AITD remains insufficiently characterized and requires further investigation. Overall, available evidence supports the need for a comprehensive assessment of T-cell subsets, including the Th1/Th2 balance, the Th17/Treg axis, and other relevant immune biomarkers (13, 17, 46). Prospective studies are warranted to establish whether such immune profiling can provide clinically relevant prognostic information beyond conventional thyroid function tests, autoantibody status, and established obstetric risk factors (**Figure 1**).

**Figure 1:**
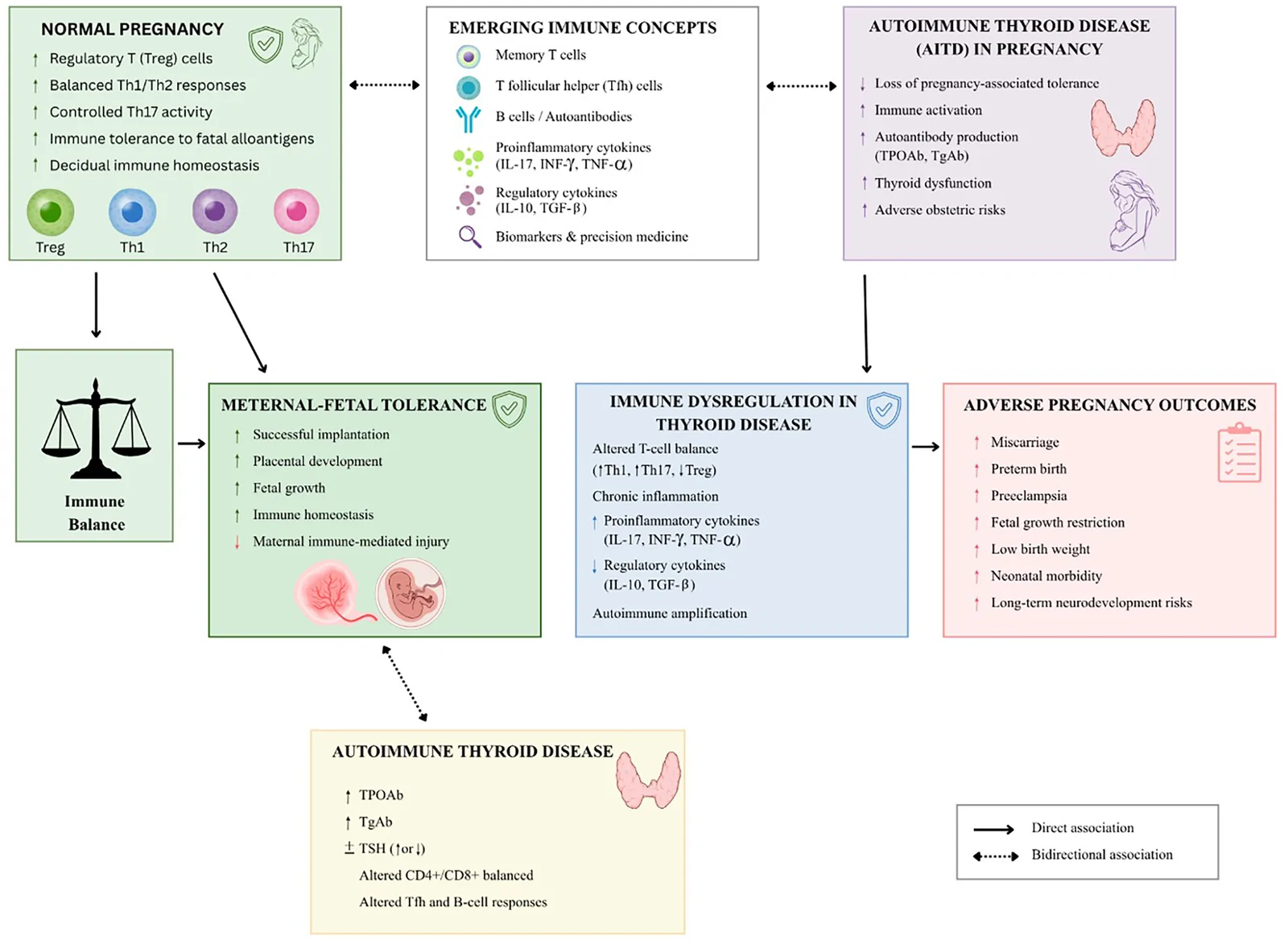
T cell - mediated immune interactions linking autoimmune thyroid disease, pregnancy, and pregnancy outcomes. Normal pregnancy is characterized by a shift toward immune tolerance that promotes maternal–fetal coexistence. Autoimmune thyroid disease (AITD) is characterized by loss of immune tolerance, immune activation, and autoantibody production and is associated with immune dysregulation and an increased risk of adverse pregnancy outcomes. T-cell subsets, B cells, autoantibodies, and cytokines interact dynamically throughout these processes. The arrows represent conceptual relationships within the proposed immunological framework and do not imply causality. Solid arrows indicate unidirectional conceptual relationships, whereas dashed double-headed arrows indicate bidirectional conceptual interactions. AITD, autoimmune thyroid disease; Treg, regulatory T cell; Th1, T helper type 1 cell; Th2, T helper type 2 cell; Th17, T helper 17 cell; Tfh, T follicular helper cell; TPOAb, thyroid peroxidase antibody; TgAb, thyroglobulin antibody; IFN-γ, interferon gamma; IL, interleukin; TNF-α, tumor necrosis factor alpha; TGF-β, transforming growth factor beta; TSH, thyroid-stimulating hormone; CD4+/CD8+, CD4-positive/CD8-positive T-cell ratio. The figure was created by the authors using Canva Pro (Canva Pty Ltd., Sydney, NSW, Australia; licensed subscription). All graphical elements were assembled and edited by the authors. Generative AI was not used to create the figure, and no copyrighted third-party material requiring additional permission was included.

## 6 Discussion

An important challenge in interpreting the available evidence is the difficulty in distinguishing the immunological effects of thyroid autoimmunity from those related to thyroid dysfunction and its treatment. Other maternal characteristics, including age, body mass index, coexisting autoimmune conditions, and previous reproductive history, may also influence both immune profiles and pregnancy outcomes (8, 9, 31, 36–39). Interpretation is further complicated by considerable heterogeneity among studies in terms of AITD phenotype, gestational age, the biological compartment examined, methods used for T-cell phenotyping, and definitions of adverse outcomes (13, 26, 27, 33, 34, 46, 47). Therefore, findings obtained in women with HT, GD, euthyroid thyroid autoantibody positivity, or overt and subclinical thyroid dysfunction should not be considered directly interchangeable (8, 9, 26, 27, 31). Although some studies have reported T-cell and cytokine alterations separately in pregnant women with HT and GD, including broadly similar directional changes in several immune parameters, the available evidence remains insufficient for a robust direct comparison of the magnitude and clinical significance of these alterations between the two phenotypes. Several limitations of the present review should also be acknowledged. As a narrative Mini Review, it was designed to provide a focused synthesis rather than a systematic or exhaustive assessment of the literature and is therefore subject to potential selection bias. In addition, the available evidence is characterized by heterogeneous study populations and predominantly observational or indirect data. Differences in thyroid functional status and treatment further limit the ability to attribute reported immune alterations specifically to thyroid autoimmunity. These limitations should be considered when interpreting the proposed relationships between T-cell dysregulation, AITD, and pregnancy outcomes. Taken together, the reported associations between T-cell imbalance and adverse pregnancy outcomes are biologically plausible, but they should not be interpreted as proof of a direct causal relationship.

### 6.1 Future perspectives

Despite increasing evidence linking T-cell dysregulation with AITD and adverse pregnancy outcomes, several aspects of immune adaptation during pregnancy remain insufficiently understood. Better characterization of dynamic changes in T-cell subsets throughout pregnancy and the postpartum period may provide novel insights into disease pathophysiology and facilitate identification of biomarkers for risk stratification and targeted therapeutic approaches. Because maternal age, body mass index, and parity may influence baseline gestational immune profiles, future prospective cohort studies should systematically record and adjust for these clinical covariates to minimize potential confounding.

Particular attention should be directed toward the Th17/Treg axis and pregnancy-induced memory T-cell populations, which may contribute to maternal–fetal tolerance and postpartum immune regulation (52, 55). Advances in immune profiling technologies may further improve understanding of immune interactions at the maternal–fetal interface and contribute to the development of personalized monitoring strategies and novel immunomodulatory approaches aimed at improving maternal and fetal outcomes.

## 7 Conclusion

Pregnancy requires highly coordinated and dynamic immune adaptations that ensure maternal–fetal tolerance while preserving protective immune function. T-cell subsets, particularly Th1, Th17, and regulatory T cells, play central roles in maintaining this immunological balance throughout gestation (3, 25). AITD is characterized by disturbances in T-cell–mediated immune regulation, including enhanced pro-inflammatory responses and impaired immune tolerance (4, 27, 30). During pregnancy, these immune abnormalities may interfere with physiological maternal adaptation and may be associated with adverse pregnancy outcomes, including pregnancy loss, preterm birth, and preeclampsia, as well as postpartum thyroid dysfunction (36–39, 44, 50). Available evidence suggests that alterations in the Th17/Treg axis warrant further investigation as a potential immunological link between thyroid autoimmunity and pregnancy complications (13, 46, 47). Importantly, these findings should not be interpreted as indicating identical T-cell dysregulation across AITD phenotypes, and further phenotype-specific studies are required to define the immunological characteristics of HT and GD during pregnancy. Further elucidation of the underlying immune mechanisms may help identify candidate biomarkers, improve identification of women at increased risk of adverse pregnancy outcomes and support the development of more individualized management strategies in AITD. However, current evidence is insufficient to establish causality or support routine clinical measurement of T-cell subsets.
